# Student Longevity

**Published:** 1858-10

**Authors:** 


### STUDENT LONGEVITY.

STUDENTS are not necessarily short-lived. There is nothing in the active exercise of the brain which impairs the constitution, or lessens the duration of existence. NEWTON died at the age of eighty-five; ROGER BACON reached his eightieth year; and his namesake, the Lord High Chancellor of England, of whom it was said that, "as a man of genius and a philosopher, no language can be too lofty for his praise," was in his sixty-sixth year when he closed his eyes; and but for his extravagant habits, might easily have lived a quarter of a century longer; COPERNICUS was seventy. These are among the very greatest names in science, philosophy, and law, of the era preceding our own; and of the great minds of the present generation which dazzle the eye by the splendor of their shining—by the extent of their attainments, or the beauty of their characters, and often both—we might name, in medicine, CHARLES CALDWELL, of Kentucky, aged eighty-one; in genius and learning, ELIPHALET NOTT, now in his eighty-sixth year; in Natural Philosophy, Professor SILLIMAN, but six years younger. In his ninetieth year, the great HUMBOLDT is not conscious of any abatement of mental power—his splendid mind still commands the veneration of two hemispheres. The greatest students among our political men were, BENTON, at seventy-five; and JOHN QUINCY ADAMS died with his harness on, at the age of fourscore years. HAMEL, one of the greatest scientific minds of Russia, visits Valentia, "not far from ninety," to wonder and admire, as he gazes at the accomplishment of the age—the Atlantic Cable. Then, coming down to a broad fact here at home among ourselves—within the past year, of thirty graduates of Harvard College dying, more than one-half were over seventy years; nearly a quarter were over eighty; and one died at the age of ninety-three—during the same year, of one-half the graduates of old Yale who have passed to fields of exploration beyond the River of Death, and to them, all new, one-half had passed the limits of threescore years and ten. When it is taken into account, that of all the modern names given, it is known that a temperate life has been a peculiarity, almost to a proverb—temperate as to drinking and as to eating—almost abstemious—we are impelled to the conclusion, that a man who is temperate as to the habits of his life, may study never so hard, and not only "endure to the end" of the usual limit of "threescore years and ten;" but even at that age may possess a mind "undimmed by the flight of years." On the other hand, when we see a great mind go out in the night of the grave at forty or fifty, or any short of threescore, that mind should at least inquire, and leave an answer as a beacon-light for after-voyagers: Do I die thus early "in the course of Nature," or has it come thus by mine own hand, in that I have not, as I ought to have done, striven against the passions and appetites of a lower nature? To die at the maturity of a great intellect—upon the very entrance of fields of view, just expanding to the enraptured gaze of the beholder—the loss, to himself of a pure delight, how immeasurable! and to the world, figures may not compute it. Doubtless, the dial of "progress" has been put back many a degree in just such way as this. Reader! health is a duty to yourself and the age you live in. The greater your intelligence, the greater your dereliction; and for which you will have to account at the Judgment. To know what health is, and how to preserve it, is the great object of our writings; and the regret is, that it is a kind of knowledge almost entirely neglected, by high and low, rich and poor together, until a time in life too late to make its acquisition of any great practical advantage—a knowledge which the fewest of the few are wise enough to acquire in early life. None but an ADAMS, a NOTT, a BENTON, a HUMBOLDT in embryo, is competent to a wisdom like this.

